# Exacerbation of previously undetected ulcerative colitis in a patient undergoing nivolumab for metastatic melanoma: A case report

**DOI:** 10.1097/MD.0000000000044833

**Published:** 2025-09-26

**Authors:** Je-Seong Kim, Chae-June Lim, Young-Eun Seo, Chan-Muk Im, Joo Yeon Koo, Hyung-Hoon Oh, Young-Eun Joo

**Affiliations:** a Department of Internal Medicine, Chonnam National University Medical School, Hwasun, South Korea; b Department of Pathology, Chonnam National University Hwasun Hospital and Medical School, Hwasun, South Korea.

**Keywords:** case report, immune checkpoint inhibitor, metastatic melanoma, nivolumab, ulcerative colitis

## Abstract

**Rationale:**

In patients receiving immune checkpoint inhibitors, subclinical inflammatory bowel disease can be unmasked, leading to severe colitis. Ulcerative colitis, in particular, may remain undetected when initial symptoms are mild. This report describes a patient with metastatic melanoma whose previously unrecognized ulcerative colitis flared severely after initiating nivolumab.

**Patient concerns:**

A 35-year-old man presented with chronic mild diarrhea, persisting for several months, which was initially overlooked. Three months after starting nivolumab therapy, he developed frequent diarrhea and hematochezia that required hospitalization.

**Diagnoses:**

Sigmoidoscopy demonstrated ulcerations with bleeding in the rectosigmoid junction and rectum. Histopathology revealed crypt architectural distortion and inflammatory infiltrates, consistent with ulcerative colitis. Infectious etiologies were excluded.

**Interventions:**

High-dose intravenous corticosteroids were administered, followed by an oral steroid taper and 5-aminosalicylic acid. Nivolumab was briefly held during acute exacerbations but was ultimately resumed to continue melanoma management.

**Outcomes:**

Two months after resuming nivolumab, the patient experienced a diffuse ulcerative colitis flare, now involving the entire colon. A second course of high-dose pulse steroid therapy was initiated, and a further hold on nivolumab is currently under consideration.

**Lessons:**

Mild ulcerative colitis can be unmasked and exacerbated by immune checkpoint inhibitors such as nivolumab, emphasizing the importance of early evaluation for inflammatory bowel disease in patients with persistent diarrhea.

## 1. Introduction

Immune checkpoint inhibitors (ICIs) play a pivotal role in cancer therapy.^[1]^ In particular, PD-1 (programmed cell death protein 1) blockers such as nivolumab have transformed treatment paradigms for metastatic melanoma by enhancing T-cell–mediated tumor control.^[2,3]^ However, these agents may precipitate immune-related adverse events affecting various organ systems, including the gastrointestinal tract.^[4]^ Numerous reports have documented exacerbation of preexisting inflammatory bowel disease following ICI treatment.^[5,6]^ In patients with previously undetected inflammatory bowel disease – such as ulcerative colitis – immune activation can also provoke severe or recurrent colitis flares.^[7]^

Here, we report the case of a 35-year-old man with metastatic melanoma who experienced 2 severe colitis exacerbations after initiating nivolumab, revealing underlying ulcerative colitis that had previously been masked by mild symptoms. We discuss the diagnostic considerations, management strategies, and the challenge of balancing optimal cancer control with the need to mitigate gastrointestinal toxicity.

## 2. Case presentation

A 35-year-old male had been undergoing treatment for metastatic melanoma for 9 years, including multiple surgical procedures (wide local excisions, lymph node dissections, and wedge resections for pulmonary metastases) as well as systemic therapies (interferon-alpha and targeted agents). Follow-up imaging with an F-18 FDG PET-CT (fluorodeoxyglucose positron emission tomography–computed tomography) demonstrated hypermetabolic activity in the rectosigmoid colon. Subsequent colonoscopy revealed features consistent with ulcerative proctitis. At that time, ASCA (anti-Saccharomyces cerevisiae antibody) immunoglobulin G was positive, and the fecal calprotectin level was markedly elevated at 2671.0 mg/kg, further supporting the diagnosis of ulcerative colitis. However, the biopsy results were inconclusive for ulcerative colitis and instead suggested a possible extranodal marginal zone B-cell lymphoma of mucosa-associated lymphoid tissue. While the patient had mild diarrhea, he did not mention it because he lacked more concerning symptoms such as abdominal pain or hematochezia, and the clinicians did not specifically ask about gastrointestinal manifestations. Consequently, ulcerative colitis was not strongly suspected, the patient was not referred to the gastroenterology division for further management, and no additional evaluation with fecal calprotectin was performed.

Progression of melanoma severity prompted a switch to nivolumab 3 months earlier (August 20, 2024). He had reported mild diarrhea (three to 4 loose stools/day) for approximately 3 to 6 months prior to initiating ICI therapy.

## 3. First severe flare

After 6 cycles of nivolumab therapy, the patient developed frequent bloody diarrhea (up to 10 stools/day), lower abdominal cramping, and fatigue. Physical examination revealed mild diffuse tenderness without peritoneal signs. Laboratory investigation showed leukocytosis (11,700/mm^3^) and a slight elevation in C-reactive protein (0.46 mg/dL), while the hemoglobin level remained within the normal range. Procalcitonin and stool cultures were not assessed.

Colonoscopy demonstrated continuous mucosal erythema and ulcerations from the rectum to the rectosigmoid junction (Mayo 2–3). Biopsies revealed cryptitis and inflammatory infiltrates consistent with ulcerative colitis.

## 4. Interventions and outcome

The patient was treated with 5-aminosalicylic acid (5-ASA) at 4 g/day and high-dose intravenous corticosteroids (methylprednisolone 60 mg/day), resulting in rapid symptom improvement. He was discharged on a tapered course of oral steroids over 4 weeks. The 5-ASA at 4 g/day was continued as maintenance therapy. Given the urgent need to control metastatic melanoma, nivolumab was resumed following symptom resolution.

## 5. Second severe flare

**Figure 1.**
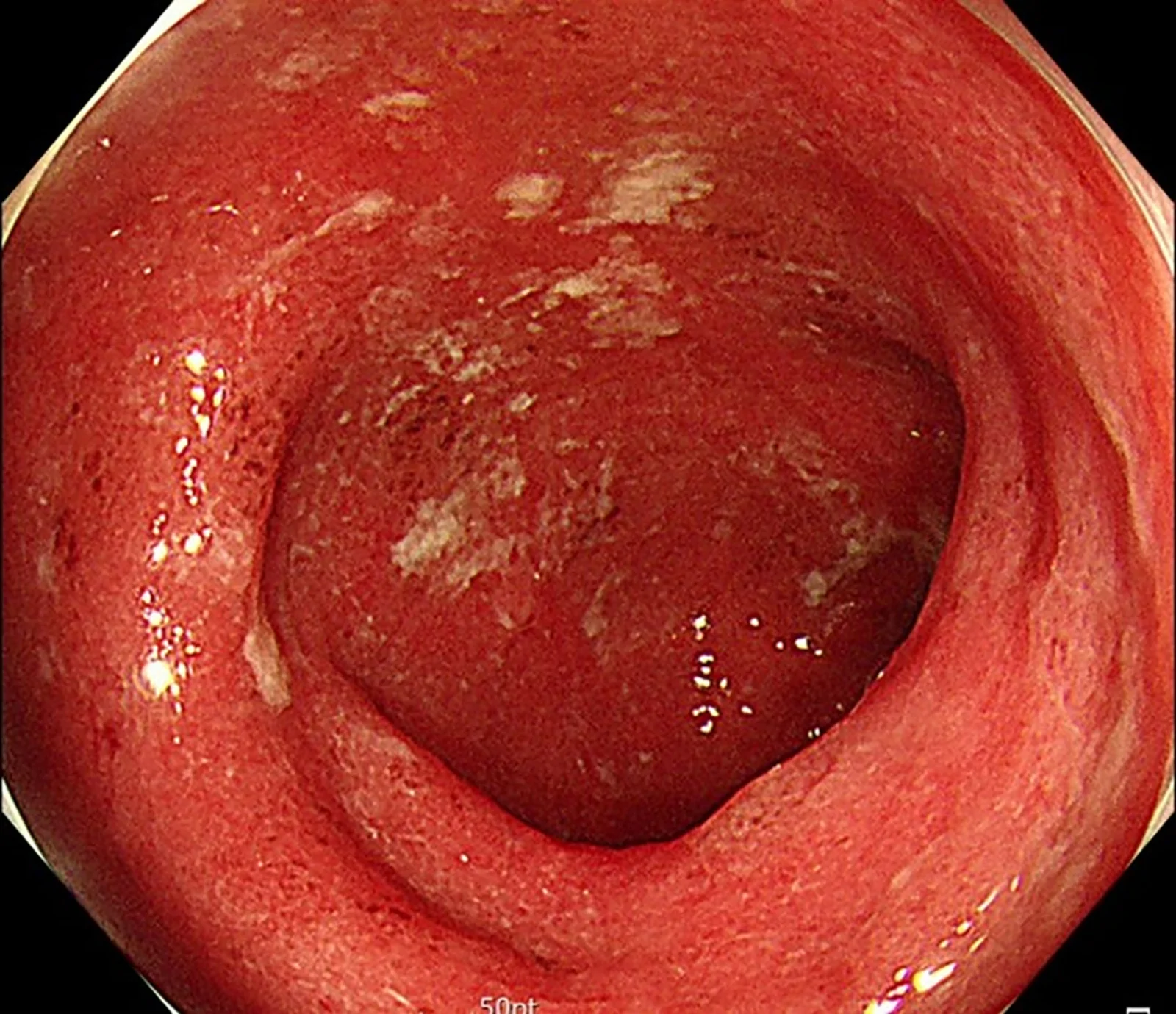
Mucosal erythema, erosions, and absent vascular pattern throughout the entire colon on colonoscopy (Mayo 2).

**Figure 2.**
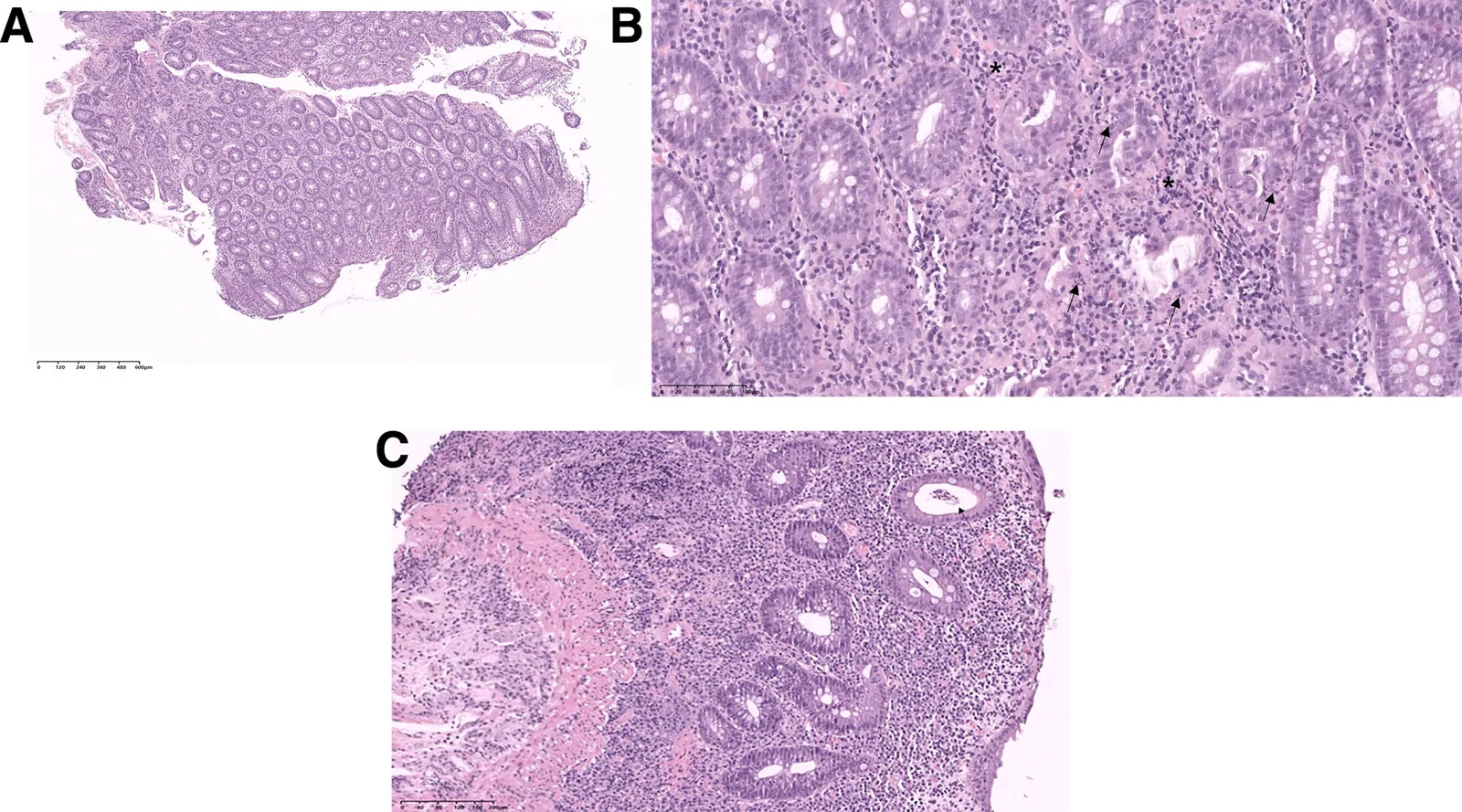
Microscopic findings of colorectal specimen. (A) Lymphoplasmacytic infiltration with prominent neutrophil infiltration and cryptitis (40×). (B) Prominent neutrophil infiltration (asterisk) and cryptitis (arrows) (200×). (C) Cryptitis in most glands (short arrows), accompanied by crypt architectural distortion. A crypt abscess is identified in 1 gland (long arrow) (100×).

After an additional 5 cycles of nivolumab, the patient again presented with 8 to ten loose stools/day and hematochezia. Repeat endoscopic evaluation showed diffuse colonic inflammation (Mayo 2), and histopathology again confirmed active ulcerative colitis. Stool studies were negative for infection (Figs. 1 and 2).

## 6. Interventions and outcome

High-dose intravenous corticosteroids (methylprednisolone 60 mg/day) were reinitiated, yielding a rapid clinical response. After 3 weeks, the steroids were tapered, and maintenance therapy with 5-ASA at 4 g/day was continued. Nivolumab was discontinued because of recurrent ulcerative colitis flares. We are now considering reintroducing dabrafenib and trametinib, commonly used for BRAF-mutant melanoma, which were previously discontinued due to recurrent joint infections.

## 7. Discussion

This case illustrates how previously undiagnosed ulcerative colitis can present as severe colitis flares once immune checkpoints are inhibited. While ICI-induced colitis can develop in patients without prior gastrointestinal disease, underlying inflammatory bowel disease may also become unmasked and exacerbated as T-cell activity increases.^[5]^ In our patient, mild diarrhea that predated ICI therapy progressed to 2 acute flares shortly after starting nivolumab, underscoring the importance of comprehensive gastrointestinal evaluation in patients with persistent symptoms both before and during therapy.

It can be challenging to differentiate ICI-induced colitis from unmasked ulcerative colitis because both conditions exhibit overlapping endoscopic (mucosal ulceration) and histopathologic (cryptitis, plasma cell infiltration) features.^[8]^ Owing to the widespread use of ICIs, the notion of these unmasked inflammatory bowel disease has come to the fore. Unmasked inflammatory bowel disease refers to a preexisting but previously undiagnosed condition that becomes apparent after being triggered by an ICI-related flare. In contrast, ICI-induced colitis denotes a new-onset colitis that develops only after initiation of ICI therapy.^[9]^ Colitis mimicking ulcerative colitis after immune checkpoint inhibition has been reported and typically responds to standard ulcerative colitis treatments, such as steroids and mesalamine.^[10]^ This resemblance adds to the difficulty in distinguishing such cases from previously unrecognized or latent ulcerative colitis.^[11]^

In our case, differentiating between ICI-induced colitis and unmasked ulcerative colitis was challenging; nevertheless, the patient’s history of subclinical chronic diarrhea prior to nivolumab initiation, in conjunction with elevated calprotectin levels, provides compelling evidence for previously unrecognized ulcerative colitis that was subsequently exacerbated by immunotherapy. Additionally, a colonoscopy report obtained 1 month prior to the first dose of nivolumab described erythematous mucosa, shallow erosions, and a loss of vascular markings in the rectum – findings suggestive of ulcerative proctitis that were initially overlooked by some clinicians. Therefore, thorough medical record review is essential for differentiating between ICI-induced colitis and ulcerative colitis flares. Moreover, a recent study reported that, unlike immune-related colitis, unmasked inflammatory bowel disease demonstrates histologic features of chronicity, including architectural distortion and basal lymphoplasmacytic infiltration.^[7]^ The pathologic report of our patient demonstrated these chronicity features, consistent with unmasked inflammatory bowel disease, which also help distinguish it from typical ICI-induced colitis.

The mechanisms underlying the aggravation of inflammatory bowel disease remain unclear. However, it has been proposed that gut bacteria influence gastrointestinal mucosal immunity, while persistent T-cell activation contributes to a pro-inflammatory state.^[12,13]^ CTLA-4 (cytotoxic T-lymphocyte–associated protein-4) blockade appears to disrupt gut homeostasis more severely than PD-1 or PD-L1 (programmed death-ligand 1) antibodies, but recent studies suggest that anti–PD-1/PD-L1 treatment is also associated with immune-mediated enterocolitis in inflammatory bowel disease patients.

Corticosteroids remain the first-line therapy for moderate-to-severe gastrointestinal toxicity related to ICIs or acute ulcerative colitis exacerbations.^[4,14]^ Our patient responded promptly to steroid treatment during both flares, facilitating rechallenge with nivolumab – a critical step for controlling metastatic melanoma. If repeated steroid-refractory flares occur, second-line immunosuppressive agents (infliximab or vedolizumab) may be considered.^[14]^ In melanoma patients, however, there is a theoretical concern about the use of anti–tumor necrosis factor-alpha therapy, as tumor necrosis factor-alpha had previously been used for regional therapy of melanoma. This concern has prompted some clinicians to favor vedolizumab for gut-selective immunosuppression.^[15]^

The decision to continue nivolumab hinges on the severity and frequency of colitis flares versus the need for disease control in metastatic melanoma. Current guidelines on immune-related adverse events recommend resuming ICIs after the resolution of symptoms and consider PD-1/PD-L1 agents to be safer than CTLA-4 inhibitors.^[14]^ In our case, the patient’s robust response to steroids enabled the continuation of PD-1 blockade. Given the potential for ulcerative colitis exacerbation following the reinitiation of nivolumab, ongoing follow-up with fecal calprotectin measurements, repeat endoscopy, and close interdisciplinary collaboration is essential for the early detection of colitis exacerbation.

## 8. Conclusion

Previously unrecognized ulcerative colitis can be unmasked by PD-1 inhibitors, resulting in significant gastrointestinal toxicity in patients treated for metastatic melanoma. Early gastrointestinal evaluation for persistent diarrhea, judicious use of corticosteroids for flares, and a multidisciplinary approach are key to optimizing outcomes for both cancer control and colitis management. If repeated flares or steroid refractoriness occur, vedolizumab might be a viable option to minimize systemic immunosuppression while permitting ongoing immunotherapy.

## Author contributions

**Investigation:** Je-Seong Kim, Chae-June Lim, Young-Eun Seo, Chan-Muk Im.

**Writing – original draft:** Je-Seong Kim, Hyung-Hoon Oh.

**Writing – review & editing:** Je-Seong Kim, Young-Eun Joo.

**Data curation:** Joo Yeon Koo.

**Conceptualization:** Hyung-Hoon Oh.

**Resources:** Hyung-Hoon Oh, Young-Eun Joo.

**Supervision:** Young-Eun Joo.
